# Regulatory dendritic cells: there is more than just immune activation

**DOI:** 10.3389/fimmu.2012.00274

**Published:** 2012-09-04

**Authors:** Susanne V. Schmidt, Andrea C. Nino-Castro, Joachim L. Schultze

**Affiliations:** Genomics and Immunoregulation, LIMES-Institute, University of BonnBonn, Germany

**Keywords:** cancer, chronic infection, chronic inflammation, regulatory dendritic cells, IDO

## Abstract

The immune system exists in a delicate equilibrium between inflammatory responses and tolerance. This unique feature allows the immune system to recognize and respond to potential threats in a controlled but normally limited fashion thereby preventing a destructive overreaction against healthy tissues. While the adaptive immune system was the major research focus concerning activation vs. tolerance in the immune system more recent findings suggest that cells of the innate immune system are important players in the decision between effective immunity and induction of tolerance or immune inhibition. Among immune cells of the innate immune system dendritic cells (DCs) have a special function linking innate immune functions with the induction of adaptive immunity. DCs are the primary professional antigen presenting cells (APCs) initiating adaptive immune responses. They belong to the hematopoietic system and arise from CD34^+^ stem cells in the bone marrow. Particularly in the murine system two major subgroups of DCs, namely myeloid DCs (mDCs) and plasmacytoid DCs (pDCs) can be distinguished. DCs are important mediators of innate and adaptive immunity mostly due to their remarkable capacity to present processed antigens via major histocompatibility complexes (MHC) to T cells and B cells in secondary lymphoid organs. A large body of literature has been accumulated during the last two decades describing which role DCs play during activation of T cell responses but also during the establishment and maintenance of central tolerance (Steinman et al., 2003). While the concept of peripheral tolerance has been clearly established during the last years, the role of different sets of DCs and their particular molecular mechanisms of immune deviation has not yet fully been appreciated. In this review we summarize accumulating evidence about the role of regulatory DCs in situations where the balance between tolerance and immunogenicity has been altered leading to pathologic conditions such as chronic inflammation or malignancies.

## Major functional states of DCs during immune activation

A major focus of research into DC biology was built on observations during immune activation. DCs are a heterogeneous cell population that can acquire diverse maturation states and functions. Intensive studies of DC development in mice lead to the conception that DCs derive under homeostatic conditions from hematopoietic CD34^+^ stem cells in the bone marrow and potentially in the intestinal lamina propria (Bogunovic et al., 2009) while monocyte-derived DCs have been described under inflammatory conditions (Cheong et al., 2010). Also, human CD14^+^ CD34^+^ PBMCs were described to give rise to DCs by the influence of platelet endothelial cell adhesion molecule-1 (Ferrero et al., 1998). Myeloid precursor cells in the bone marrow give rise to common DC precursor cells with the ability to proliferate and relocate to bone marrow, spleen, and lymph nodes. The major function of DCs has been attributed to the initiation steps of immune activation leading to protection of the individual against invading pathogens and the immune surveillance against transformed cells.

At least two categories of DCs have been described for the mammalian immune system (Banchereau et al., 2000). Myeloid DCs (mDCs) also called conventional DCs have a strong capability to capture antigens which enables them to stimulate T cells. These major antigen presenting and activating cells comprise a very heterogeneous group of cells expressing high levels of MHC class II and integrin CD11c on their cell surface, but also other adhesion molecules, like LFA-1 (CD11a), LFA-3 (CD58), ICAM-1 (CD54), ICAM-2 (CD50), and ICAM-3 (CD102). The costimulatory molecules CD80 and CD86 have been established as hallmarks of DC maturation during an immune response with CD86 being expressed at early stages of maturation, while CD80 (and also CD83) become upregulated in mature DCs. The Langerhans cells of the skin are one major representative subgroup of the mDCs continuum. Murine mDCs are characterized by the expression of CD11b and CD11c and are generated *in vitro* by stimulation of bone marrow progenitor cells while in the human, DCs are often generated from peripheral blood monocytes using GM-CSF and IL-4 (Sallusto and Lanzavecchia, 1994).

A second group of DCs are plasmacytoid DCs (pDCs) that are found in circulation and in peripheral lymphoid organs. In comparison to other APC the capacity of pDCs to present antigens is rather low since immature pDCs express only low levels of MHC-II or other costimulatory molecules. Upon activation they secrete large amounts of IFNα and IFNβ (Cella et al., 1999; Siegal et al., 1999). Infection with RNA- and DNA-viruses induces IFN-related immune responses in pDCs human and mice after the recognition of viral genomes via pattern recognition receptors (PRR) such as toll-like receptors (TLRs) 7 and 9 (Lund et al., 2003; Di Domizio et al., 2009; Swiecki and Colonna, 2010). Characterization via surface receptors revealed that pDCs do not express markers commonly present on human mDCs such as CD11c, but express instead the interleukin 3 receptor (CD123) and exclusively the type II c-type lectin BDCA-2 (CD303) which is involved in the presentation of antigens to T cells (Dzionek et al., 2001). In contrast to human pDCs murine pDCs are characterized by the expression of CD11c, B220, Gr-1, CD45RA, Ly49Q, BST2, and Siglec-H (Gehrie et al., 2011). It is assumed that these cells play a major role in anti-viral immune responses since they produce high amounts of IFNα after viral infection.

A third group named follicular DCs (fDCs) can be found in the germinal centers of lymph nodes presenting antigens to B cells to maintain immune memory. fDCs extracted out of human tonsils have been found to express the surface receptors CD21, CD23 CD35, and cell cycle markers DRC-1, Ki-M4 or DR53 (Kim et al., 1994). Interestingly, in contrast to pDCs and mDCs fDCs share some common antigens such as 3C8 with fibroblasts suggesting that these cells share some molecular programs (Lindhout et al., 1999; Lee and Choe, 2003; Vinuesa et al., 2010).

**Figure 1:**
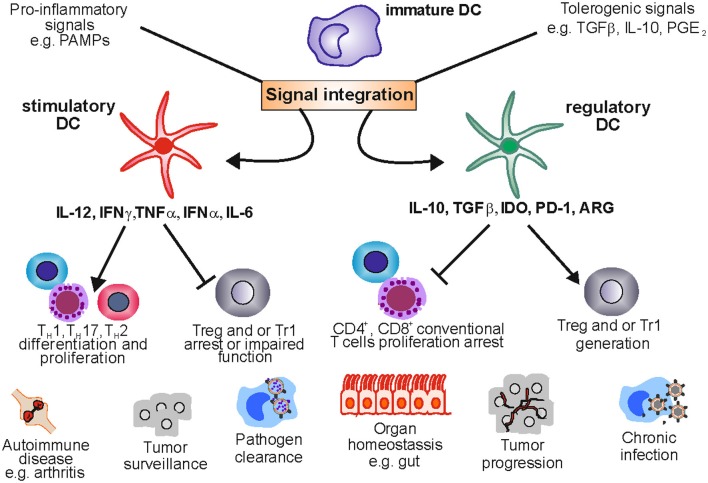
**Stimulatory and regulatory dendritic cells in health and disease**. DCs are a plastic lineage able to process and integrate signals from the microenvironment. Under pro-inflammatory conditions stimulatory DCs promote an effective immune response by stimulating T cell proliferation and shaping T cell responses toward TH 1, TH 2, or TH17 phenotypes. This crucial role allows the immune system to clear pathogens and keep transformed cells in check. Nevertheless, uncontrolled DC activation can lead to tolerance ablation, fostering the development of autoimmune diseases like rheumatoid arthritis. Under a tolerogenic environment DCs acquire regulatory functions suppressing T cell activation and proliferation and providing signals that enable Treg and Tr1 differentiation and expansion. This function maintains tolerance in organs like the gut which are exposed to a variety of harmless antigens. However, DCreg function can be exploited by tumors and pathogens leading to tumor progression and chronic infection.

Immature DCs patrol via the blood systems throughout the body and can invade peripheral tissues to take up antigens from infected or dying cells via macropinocytosis, phagocytosis, and endocytosis (Steinman et al., 1999). Migration of DCs from peripheral tissues to lymph nodes also occurs under steady state conditions in absence of infection and might contribute to tolerance induction. Receptors of the C-type lectin family like DEC205, DCIR or the mannose receptor (CD206) directly capture antigens and direct them to antigen processing antigen processing machinery in the endosomal compartment or the cytosol (Villadangos and Schnorrer, 2007). The expression of PRR including TLRs, NOD-like receptors and RIG-like helicases by DCs enables these immune cells to recognize bacterial (e.g., LPS) or viral (e.g., single-stranded RNA) compounds, so called pathogen associated molecular patterns (PAMPs) (Janeway and Medzhitov, 2002). More recently, it was shown that DCs also recognize intracellular host factors released to the extracellular space after cell damage, called damage associated molecular patterns (DAMPs) like HMGB1 or S100A/B proteins. mDCs are found to express TLR1, TRL2, TRL4, TLR5, and TLR8, while pDCs express TLR-7 and TLR-9. After activation of the TLR-signaling cascade via the adaptor molecules MYD88 and TRIF pro-inflammatory transcription factors like NFκB and several interferon regulating factors (IRFs) are activated and lead maturation of DCs and to the expression of immune activating mediators (Hemmi and Akira, 2005). A central contribution to immune activation is the presentation of processed antigens via major histocompatibility complexes (MHC) in presence of costimulatory molecules on the cell surface of DCs to T cells and B cells in secondary lymphoid organs. The transport of peptide-loaded MHC molecules to the cell surface is accompanied by an increased expression of costimulatory molecules like CD80 and CD86. Other typical maturation marker on matured human and mice DCs are elevated levels of HLA-DR, CD40, CD80, CD1a, and CD54 (Reis e Sousa, 2006). The activation of signaling cascades downstream of PRRs also induces DC migration to afferent lymph nodes to present antigens to T cells and B cells (Hemmi and Akira, 2005) which is mediated by the chemokine and homing receptor CCR7 along a gradient of the two chemokines CCL19 Epstein-Barr virus-induced molecule-1 ligand chemokine (ELC), and CCL21 Secondary lymphoid tissue chemokine (SLC) (Sanchez-Sanchez et al., 2004; Riol-Blanco et al., 2005). Mature mDCs secrete high amounts of inflammatory cytokines such as IL-12, necessary for the differentiation of naïve T cells toward certain T helper cell subsets (Figure 1). In principle, DCs unite at least four different signals in the immune synapse. One activation signal is established by the presentation of processed antigens via MHC molecules that interact with the T cell receptor complex. The binding of the adhesion molecule ICAM-1 with LFA-1 on the T cell surface strengthens the contact of both immune cells and the so-called signal two is established by the interaction between costimulatory molecules CD80/CD86 expressed on matured DCs with CD28 on T cells. Finally, an interaction between CD40 on DCs and CD40L on T cells is established leading to increased IL-12 production of DCs. The elevated levels of IL-12 finally lead to a T_H_1 polarization and secretion of the cytokine IFNγ which is necessary for the recruitment of macrophages and cytotoxic T cells. While there is no doubt about the exceptional role of DCs during immune activation, more and more evidence has been accumulated demonstrating that these specialized cells can also exert regulatory functions.

## Defining regulatory dendritic cells

While activation and maturation of dendritic cells (DCs) and their immunostimulatory capacity are clearly linked to a distinct phenotypic change with upregulation of MHC molecules, costimulatory molecules, and the enhanced production of inflammatory cytokines, this is less clear for DCs exerting regulatory functions. Initially, regulatory, inhibitory or even tolerance-inducing capabilities were assigned to immature DCs, a differentiation state prior maturation. It has been a common view that DCs exist in an immature and a mature state. Initial experiments demonstrated that immature DCs can induce tolerance, which was explained by the finding that immature DCs process and present antigens in the absence of costimulation, leading to T cell anergy and deletion (Jonuleit et al., 2000; Lutz et al., 2000; Reis e Sousa, 2006; Manicassamy and Pulendran, 2011). However, during the last years, fully matured DCs with regulatory functions have been observed in numerous distinct settings suggesting that regulatory DCs (DC_reg_) are a functional state rather than a unique subpopulation defined by phenotypical markers. For example, in a murine colitis model CD103^+^ DCs acquire immune-activating functions under inflammatory conditions and express pro-inflammatory cytokines (Laffont et al., 2010). However, under steady state conditions CD103^+^ DCs in the gut where shown to be strong inducers of T cell tolerance, which was manifested by their capacity to induce Foxp3^+^ T_reg_ from CD4^+^ naïve precursors (Del Rio et al., 2010; Scott et al., 2011). A similar situation was observed in the liver where low numbers of pDCs are associated with viral persistence during chronic hepatitis C infection while elevated numbers of highly active DCs are associated with pathogen clearance (Lai et al., 2007). At the same time resident pDCs in the liver promote immune regulation through various mechanisms (Matta et al., 2012). Yet another example is the coexistence of DCs with stimulatory and regulatory functions in the tumor microenvironment. Depending on the expression of immunomodulatory factors and cytokines in the tumor, DC_reg_ together with other immunoregulatory cells can be recruited to the tumor environment (Shurin et al., 2011; Gabrilovich et al., 2012). Further evidence for the existence of fully matured DC_reg_ came from a murine asthma model, demonstrating that fully matured DCs expressing high levels of costimulatory molecules stimulated T_reg_ development via an IL-10 depending mechanism (Akbari et al., 2001). In humans, monocyte-derived DC stimulated with prostaglandin E2 (PGE_2_) and TNFα exhibit a fully mature phenotype characterized by high expression of costimulatory molecules and pro-inflammatory cytokines, yet they suppress T cell activation via a combination of factors like indoleamine 2,3 deoxygenase (IDO) and IL-10 (Popov et al., 2006, 2008; Von Bergwelt-Baildon et al., 2006). Furthermore, it was shown that DCs with intermediate features between the immature and mature state expressing costimulatory molecules but only low levels of inflammatory cytokines, such as IL-12, IL-6, and TNFα, are also characterized by regulatory function (Lutz and Schuler, 2002). Moreover, the potential therapeutical application of semi-mature DCs as tolerance promoters have been recently reviewed (Lutz, 2012).

In summary, there seems to be significant heterogeneity of DC populations with regulatory function, which might be due to the plasticity of DCs capable of reacting to and integrating environmental signals from different microenvironments. Nevertheless they share the ability (1) to regulate or inhibit T cell activation, and (2) to induce and promote T_reg_ development and expansion (Figure 1).

## Anti-inflammatory mediators can drive DCs toward regulatory function

Since regulatory function of DCs is linked to environmental cues several soluble factors such as TGFβ, IL-10, or PGE_2_ known to play a role in immune inhibition have been linked to the induction of regulatory DCs (Popov and Schultze, 2008). Even under steady state conditions, these factors play an important role for the integrity of many organ systems, particularly those with close contact to the outside world such as lung or intestine. For example the intestine is in constant contact with a large variety of antigens and it is of vital importance to discriminate between harmless nutrients, commensal flora, and potential threats (Iweala and Nagler, 2006). TGFβ is produced by intestinal epithelial cells thereby fostering the generation of DC_reg_ and subsequently T_reg_ cells. Neutralization of TGFβ directly leads to a diminished DC_reg_ capacity to induce T_reg_ cells (Iliev et al., 2009). Along the same lines, Belladonna and coworkers demonstrated that CD8^+^ pDC rely on autocrine TGFβ stimulation but also IDO to keep tolerance under steady state conditions. Moreover, they showed that CD8^−^ immunogenic DCs do not produce TGFβ and yet externally added TGFβ induces IDO changing immunogenic DC into DC_reg_ (Belladonna et al., 2008). TGFβ also plays an important role for immunosuppression in the brain, which at least in part is also due to regulatory functions of DCs in this compartment. In a recent report, it could be demonstrated that blockade of TGFβ receptor signaling in DCs caused severe autoimmune encephalitis indicating an important role of this factor in DCs to maintain tolerance (Laouar et al., 2008). Another well-established factor inducing immunoregulatory functions in DCs is interleukin-10 (IL-10). DCs exposed to IL-10 fail to induce immunostimulatory cytokines such as IL-12 (De Smedt et al., 1997) or TNFα. Moreover, IL-10 prevents the upregulation of immunostimulatory molecules such as MHC class II and CD86. The quintessence is an impaired ability of IL-10 primed DCs to induce allogeneic T cell responses (Jonuleit et al., 2000; Moore et al., 2001; Pletinckx et al., 2011). Since the functional outcome of DCs depends on the exogenous signals integrated by these cells, it is not surprising that some factors can have both immunostimulatory as well as regulatory functions, depending on signal strength, time of exposure, and combination with other factors. The effect of PGE_2_ exemplifies such complex interaction. During acute inflammatory immune responses PGE_2_ is widely expressed by epithelial cells, fibroblasts, and immune cells infiltrating the inflamed site (Kalinski, 2012). In such situations PGE_2_ can serve as an enhancer of the immunostimulatory response. PGE_2_ also increases CCR7 expression and is essential to promote DC migration toward the lymph node-derived chemokines CCL19 and CCL21 (Scandella et al., 2002; Legler et al., 2006). However, more recently it has been reported that the PGE_2_ effect on CCR7 expression is only transient and that these PGE_2_-treated DCs secret reduced levels of CCL19, the key chemokine attracting naïve and central memory T cells (Muthuswamy et al., 2010). During extended inflammatory responses and in chronic inflammation, PGE_2_ might deviate DCs from a stimulatory into a regulatory phenotype. Under such conditions, PGE_2_ can induce regulatory mediators such as IL-10 (Kalinski et al., 1997) or thrombospondin-1 (Doyen et al., 2003). We have previously shown that TNFα signaling in presence of PGE_2_ induces regulatory DCs expressing a myriad of inhibitory molecules such as IDO, IL-10, soluble CD25 or COX-2 further increasing PGE_2_ production (Von Bergwelt-Baildon et al., 2006; Driesen et al., 2008; Popov et al., 2008). Finally exposure of DCs to PGE_2_ leads to increased IL-12p40 secretion which is not accompanied by production of IL-12p35 leading to an overall diminished production of the bioactive IL-12 heterodimer (Kalinski et al., 2001; Von Bergwelt-Baildon et al., 2006).

As exemplified for TGFβ, IL10 or PGE_2_, DCs can integrate signals from their microenvironment in a fashion that induces regulatory rather than immunostimulatory activity by these cells. Under steady state conditions such signal integration is critical for organ homeostasis. Any changes in the balance between regulatory and immunostimulatory signals can lead to tissue pathology. While decreased regulatory capacity of DCs can be associated with enhanced inflammatory responses associated with tissue destruction, increases in DC_reg_ are linked to chronic inflammation and malignant diseases.

## Regulatory DCs in cancer

Although there is clear evidence that the immune system can eliminate malignant cells the generation of a clinically efficient immune response against cancer is a challenging task (Schreiber et al., 2011). Over the last 15 years, therapies based on the immunostimulatory capacities of DCs have been a major focus of tumor immunotherapy, yet, most clinical studies have not resulted in meaningful clinical responses (Palucka and Banchereau, 2012). A major hurdle for DC-based tumor immunotherapy is to overcome regulatory circuits within the tumor microenvironment. In addition to many other cell types with regulatory or suppressive function such as myeloid derived suppressor cells (Palucka and Banchereau, 2012), T_reg_ cells or deviated macrophages, DCs also seem to be altered in various ways in malignancies (Table 1). In some cancer types DCs are depleted from the tumor site itself but also from the circulation suggesting that these malignancies induce significant changes in DC generation (Almand et al., 2000; Gabrilovich, 2004; Satthaporn et al., 2004; Tjomsland et al., 2010). In other cancer types, DC maturation was shown to be impaired and this feature was associated with lack of T cell activation and the induction of T cell anergy thereby inducing tolerance against the tumor (Ma et al., 2012; Shurin et al., 2012). In such situations, DC maturation might not simply be blocked, but more likely DCs are deviated toward a regulatory function by integrating signals from the tumor microenvironment (Gabrilovich et al., 2012). Such tumor-associated DC_reg_ are not only able to suppress effector T cells but also induce the recruitment and expansion of T_reg_ cells.

**Table 1:** **Regulatory dendritic cells in immune diseases**.

**Disease**	**DC phenotype**	**Surface marker**	**Secreted immune modulators**	**Literature**	**Function**
Cancer	Impaired maturation/immature	Downregulation of MHCII, CD80, CD83, CD86	Missing IL-12	Aalamian et al., 2001; Gabrilovich, 2004; Yang and Carbone, 2004; Bharadwaj et al., 2007; Michielsen et al., 2011; Ma et al., 2012; Shurin et al., 2012	Induction of: T cell anergy; T cell apoptosis
	Regulatory	CD25, PD-1, B7-H1	IL-10, TGFβ, Kynurerine, sCD25 IDO, COX-2, ARG1	Toossi et al., 1990; Williams et al., 1999; Benoit et al., 2004; Rodriguez et al., 2004; Ghiringhelli et al., 2005; Chemnitz et al., 2006; Von Bergwelt-Baildon et al., 2006; Chung et al., 2009; Dumitriu et al., 2009; Krempski et al., 2011; Gabrilovich et al., 2012; Scarlett et al., 2012	
**CHRONIC INFLAMMATION**						
Systemic lupus erythematosus	pDCs	HLA-DR, CD4 (CD11c reduced)	IFNα, IL-10	Blanco et al., 2001; Lee et al., 2008; Yan et al., 2008; Jin et al., 2010	Suppression of: T cell activation; T cell proliferation; T cell function
Rheumatoide arthritis	Regulatory	CD11b, CD11c, CD18	TGFβ, BAFF, IDO	Morelli et al., 2003; Zhang et al., 2005; Kavousanaki et al., 2010	
Obesity	FFA-dependent/Regulatory (USFAs)	IL10R; Downregulation of MHCII, CD80, CD83, CD86	IL-10 (missing IL-12 secretion)	Aliberti et al., 2002; Loscher et al., 2005; Miyake et al., 2010; Draper et al., 2011	
**CHRONIC INFECTION**						
Viral infection	Impaired maturation/function (Virus-dependent)	Downregulation of CD1a, CD1b, DC-SIGN, CD80, CD83, CD86	IFNα, IL-10, IL-1β (missing IL-12, IL-6, TNFα secretion)	Kruse et al., 2000; Sarobe et al., 2003; Smed-Sorensen et al., 2004; Martinson et al., 2007; Tilton et al., 2008; Harman et al., 2011; Chentoufi et al., 2012; Dental et al., 2012; Tu et al., 2012	
Parasitical infection	Regulatory	CD11c, CD25	TNFα, IFNγ, IL-10, TGFβ, COX2, IDO, S100	Von Bergwelt-Baildon et al., 2006; Poncini et al., 2008; Popov et al., 2008; Li et al., 2011	

## Functional impairment of DCs leads to defective T cell proliferation and tolerance

At the same time when numerous clinical trials were already conducted to test the efficacy of DCs as cellular cancer vaccines it was revealed in human tumor biopsies as well as murine and rat tumor models that DCs in many malignancies present an impaired function, manifested in poor antigen processing and presentation, impaired migration and low presence of costimulatory molecules (Gabrilovich, 2004; Yang and Carbone, 2004). Early studies revealed that tumor infiltrating DCs present poor capabilities to induce T cell proliferation in an allogeneic mixed lymphocyte reaction *in vitro* (Troy et al., 1998). Consistent with these findings DCs differentiated from monocytes in presence of conditioned media derived from prostate or pancreatic cancer cell lines, showed low levels of HLA-DR, costimulatory molecules CD40, CD80, CD86, and the DC maturation marker CD83 (Aalamian et al., 2001; Bharadwaj et al., 2007). Moreover, DCs cultured in presence of human colorectal cancer explants failed to upregulate CD86 and CD80 expression in response to LPS (Michielsen et al., 2011). Other examples for immune deviation of DCs in cancer came from rat cancer models demonstrating the presence of DCs expressing low levels of costimulatory molecules that were incapable of inducing T cell activation (Chaux et al., 1997; Bonnotte et al., 2004). These and many other reports suggested that the lack of immunostimulatory function of DCs in context of malignant disease was mainly due to lack of the necessary stimulatory molecules (MHC, CD80, and CD86) which might be explained by a strong influence of this field of research by findings in T cell immunology at the same time suggesting that presence or absence of costimulation on APC is mainly responsible for decision making between T cell immunity or tolerance. However, with the identification of regulatory cells such as T_reg_ cells and the appreciation of the complexity of immunoinhibitory signals within the tumor microenvironment, this rather simple model of lack of immune function by DCs has been gradually dismissed. In fact, it is now well appreciated that DCs in cancer can acquire a spectrum of different functional states ranging from strongly immunostimulatory to regulatory or even suppressive and inhibitory states inducing T cell tolerance or even deletion. Some of the molecular mechanisms responsible for regulatory rather than immunostimulatory functions of DCs in tumor microenvironments are described in the next section.

## DCs exposed to the tumor microenvironment acquire a regulatory function

The tumor microenvironment provides many inhibitory signals that can be sensed by DCs leading to a change in their functional state. Soluble factors, such as IL-10, TGFβ, and PGE_2_ (Popov and Schultze, 2008) secreted by the tumor have been clearly linked to the induction of DC_reg_ (Table 1). Furthermore, Scarllet and coworkers showed in a murine model of ovarian cancer that the switch from immunostimulatory to immunoregulatory function of mDCs at the tumor site determined the onset of aggressive malignant tumors that escaped immune surveillance (Scarlett et al., 2012). This data suggested that the interaction of DCs within the tumor microenvironment might be relevant in the control of disease progression—at least in ovarian cancer.

Among other factors, the enzymes IDO and arginase-1 as well as TGFβ have clearly been linked to the generation and enrichment of DC_reg_ in the tumor microenvironment. IDO catalyzes the first rate-limiting step in tryptophan (Trp) degradation (Yamamoto and Hayaishi, 1967). Its activity has a dual effect leading to Trp depletion and accumulation of Trp catabolites, collectively known as kynurenines (Sugimoto et al., 2006). IDO expression has been detected in biopsies of patients with different malignancies such as esophageal (Von Bergwelt-Baildon et al., 2006), squamous cell carcinoma (McGee-Lawrence et al., 2011), non-small cell lung carcinoma (Sim et al., 2012), and melanoma amongst others (Munn and Mellor, 2007). IDO activity was linked to multiple mechanisms in immune tolerance *in vitro* as well as *in vivo*. Trp starvation and accumulation of Trp catabolites lead to T cell proliferation arrest and apoptosis (Terness et al., 2002; Von Bergwelt-Baildon et al., 2006). Furthermore, DCs can induce the expansion of autologous T_reg_ via an IDO-dependent mechanism (Chung et al., 2009). More recently, it has been reported in mice that the Trp catabolite kynurenine can bind and activate the aryl hydrocarbon receptor (AHR) on T cells leading to AHR-dependent T_reg_ generation (Mezrich et al., 2010). The induction of active allograft-specific tolerance by AHR activation has been described earlier in Balb/c mice (Hauben et al., 2008). In this study DC out of VAG539-tolerized mice induced the development of CD25^+^Foxp3^+^ T cells.

Our group has shown that mDCs cultured in the presence of TNFα and PGE_2_, two factors with high abundance in the microenvironment of many tumors (Williams et al., 1999; Benoit et al., 2004; Chemnitz et al., 2006), upregulated IDO expression concomitant with an increased expression of IL-10, COX-2, and the interleukin 2 receptor alpha chain (CD25) (Popov et al., 2006, 2008; Von Bergwelt-Baildon et al., 2006; Driesen et al., 2008). Soluble CD25 has been associated with poor prognosis in solid and hematological malignancies (Paietta et al., 1997) All these immunoregulatory mechanisms together equip DC_reg_ with powerful mechanisms to restrain T cell responses. IDO^+^ DC_reg_ can mediate apoptosis of effector T cells and at the same time promote T_reg_ cell expansion thereby shifting the balance further toward immune inhibition or suppression (Fallarino et al., 2002). Furthermore, we demonstrated that cell surface expression of CD25 and secretion of soluble CD25 act as decoy receptors for IL-2 (Toossi et al., 1990; Von Bergwelt-Baildon et al., 2006) which further inhibits T cell function. Since, IDO^+^ CD25^+^ DC_reg_ are present in the tumor microenvironment one could postulate that these regulatory cells—similar to other myeloid cells with inhibitory functions are part of the immune deviation from activation to inhibition in the tumor microenvironment. In this context, a rather alerting observation was made during a clinical vaccine trial. Patients treated with mDC based cancer vaccines showed a recruitment of IDO^+^ immune cells together with T_reg_ cells to the site of injection suggesting that under some circumstances such cellular therapies might actually enhance regulatory functions of DCs rather than potentiating immune activation (Wobser et al., 2007).

In addition to IDO^+^ mDCs, the accumulation of pDCs in tumors and tumor lymph nodes is also well documented for different malignancies including melanoma (Vermi et al., 2003) or head and neck cancer (Hartmann et al., 2003). Moreover, IDO^+^ pDCs present in murine and human tumor-draining lymph nodes, were reported to induce T cell anergy toward specific tumor antigens. Importantly, the interaction of CD80/CD86 receptors on IDO^+^ pDCs with CTLA-4 on T_reg_ cells was an important molecular interaction leading to T_reg_ cell expansion and subsequently antigen-specific anergy in effector T cells (Baban et al., 2005). More recently, Watkins and coworkers reported the presence of IDO^+^ pDCs in human and murine prostate cancer. These IDO^+^ DCs suppressed T cell proliferation and induced T cell tolerance. By gene expression profiling the authors defined a regulatory gene signature of these IDO^+^ pDCs, which was controlled by the transcription factor FOXO3 (Watkins et al., 2011).

Another enzyme that has been implicated as an immunoregulatory molecule in myeloid cells including DCs in the tumor context is arginase-1 (ARG1). ARG1 catalyzes arginine conversion into urea and L-ornithine, causing the depletion of the non-essential amino acid arginine (Munder, 2009). High ARG1 activity has been described in patients with various malignancies including gastric, colon, breast, and lung cancers (Rodriguez et al., 2004). In murine models it was reported that mDC upregulate ARG1 when exposed to different cancer cell lines. Moreover, ARG1^+^ mDC suppressed T cell proliferation via arginine depletion (Liu et al., 2009; Norian et al., 2009). In T cells L-arginine deprivation leads to an arrest of the cell cycle during G0-G1 transition (Rodriguez et al., 2007) and to reduced expression of the TCR ζ-chain (Rodriguez et al., 2004). These findings suggest that upregulation of ARG1 is yet another mechanism of DC_reg_ that impacts on the outcome of T cell activation, at least in the murine model. However, the relevance of these findings in human tumors has not yet been clarified.

A major immunosuppressive factor within the tumor microenvironment is TGFβ (Li and Flavell, 2008). It has been well documented that mDCs upregulate TGFβ expression and secrete TGFβ once exposed to tumor cell lines, e.g., non-small cell lung carcinoma cell lines (Dumitriu et al., 2009). Moreover, interaction of T cells with TGFβ producing mDCs leads to the induction of CD4^+^CD25^+^Foxp3^high^ T cells (Dumitriu et al., 2009). Consistent with these findings Ghiringhelli and coworkers showed in mouse and rat colon cancer models that DCs exposed to tumor cells could acquire the capacity to secrete TGFβ and to stimulate naturally occurring T_reg_ cells *in vivo* (Ghiringhelli et al., 2005). Therefore, expression of TGFβ is yet another mechanisms by which DCs exert regulatory rather than immunostimulatory function in the tumor microenvironment. More recently, the expression of inhibitory cell surface molecules PD-1 and B7-H1 on a subset of DCs in a murine model of ovarian cancer was linked to suppression of T cell proliferation and this effect could be abolished by blocking anti-PD-1 antibodies (Krempski et al., 2011). These data suggest that PD-1 and B7-H1 expression on DC can also contribute to immunoregulation or suppression by DCs in the tumor microenvironment. Altogether, there is accumulating evidence that the absence of T cell immunity in cancer is not solely due to the lack of expression of MHC and costimulatory molecules on DCs. Rather the existence of multiple inhibitory pathways and effector molecules in tumor-associated DCs are major mechanisms of immune deviation in cancer and DCs showing such hallmarks are part of a larger family of regulatory DCs. Further studies are necessary to delineate whether a hierarchy of regulatory pathways exists or whether these mechanisms are co-existing. This will be particularly important when developing novel strategies to overcome immunoregulation in the tumor microenvironment as a basis for the development of effective cancer immunotherapy.

## Dendritic cells in chronic inflammation

Chronic inflammation results from prolonged inflammatory immune responses that are not terminated despite the fact that the causing stimuli are often eliminated. Stimuli inducing chronic inflammatory responses vary widely ranging from numerous pathogens, self-antigens to chemical compounds. Chronic inflammatory responses are associated with diseases such as diabetes, atherosclerosis, or obesity. Another deviation from a normal immune response occurs in many autoimmune diseases and chronic exposure to allergens. Allergic responses can turn into prolonged inflammatory responses with significant changes of the inflammatory response during later time points which is similarly observed in many autoimmune diseases. In such situations, resident immune cells become continuously activated and recruit further immune cells which invade the inflamed tissue further fueling the inflammatory response. Albeit pro-inflammatory cytokines, NO, arachidonic acid metabolites and ROS are major compounds under such conditions, mechanisms exist that change the cellular programs of immune cells from immunostimulatory programs resolving inflammation to chronic inflammatory programs supporting chronification of the response. Again, myeloid cells including both macrophages and DCs play an essential role in deregulating inflammatory responses into chronic inflammation.

### Regulatory mechanisms in DCs seem to be defective in systemic lupus erythematosus

While regulatory or suppressive myeloid cells seem to play a central role for immune deviation in cancer, regulatory functions of these cells seem to be reduced or even lacking in autoimmune diseases such as systemic lupus erythematodes (SLE), rheumatoid arthritis, or psoriasis (Table 1). SLE is an autoimmune disease characterized by the loss of tolerance against self-antigens. Furthermore, an imbalance of T_H_1 and T_H_2 cells in favor of T_H_2 responses has been reported to be accommodated by decreased expression of IL-18 with unchanged expression levels of IL-10 in pDCs of SLE patients (Jin et al., 2010). Despite this dysbalance in pDCs that might favor immunoregulation when using pDCs of SLE patients in allogeneic MLR assays they failed to induce the expansion of Foxp3 expressing CD4^+^CD25^+^ T_reg_ cells (Jin et al., 2010). These data follow along the lines that activated DCs in this autoimmune disease might be incapable of suppressing effector T cell activation thereby contributing to the persistence of inflammation. Despite the lack of DCs to induce T_reg_ cells, Foxp3^+^CD4^+^CD25^+^ T cells have been reported to be elevated in peripheral blood of SLE patients, however their suppressive function seemed to be impaired which was related to high levels of IFNα secreted by antigen presenting cells (APCs) of SLE patients (Yan et al., 2008). In SLE patients pDCs seem to be a main source for IFNα. It is assumed that immune complexes containing DNA or RNA act as inflammatory stimuli maintaining the IFNα production in pDCs via TLR7 and TLR9 (Means et al., 2005; Savarese et al., 2006). However, other immune cells might also contribute to the interferon storm. For example, monocytes cultured in the presence of IFNα-rich sera from SLE patients differentiated into IFNα producing DCs (Blanco et al., 2001; Ueno et al., 2007) suggesting that a feed-forward loop exists that supports chronification of the autoimmune response in SLE. In a murine model of SLE chronification of the immune response inducing IFNα production in immature Ly6C^high^ monocytes can also be initiated by treatment with tetramehtylpentadecane (Lee et al., 2008) further supporting the hypothesis that the lack of induction of DCs with regulatory function is a major mechanisms of prolongation of exacerbated autoimmune responses in SLE patients. As a logical consequence novel therapeutic concepts for SLE are targeting at the point of induction of regulatory DCs. For example, Zhang and coworkers recently described that treatment with immune complexes lead to elevated levels of PGE_2_ secretion by FcγRIIb-overexpressing DCs which correlated with an elevated capacity to suppress T cell activation (Zhang et al., 2011). Clinically, injection of immune complex-treated FcγRIIb-overexpressing DCs into lupus-prone MRL/lpr mice was associated with prolonged survival of these mice. In another mouse model the nucleosomal histone peptide epitope H4(71-94) was used in subnanomolar doses to induce DC_reg_ which expressed TGFβ but showed reduced levels of IL-6 supporting the generation of T_reg_ cells and suppression of pro-inflammatory T_H_17 cells (Kang et al., 2007). One other therapeutical approach targeted the central pro-inflammatory transcription factor NFκB. Inhibition of this transcription factor in a murine SLE model in FcγRIIb-deficient mice with andrographolide or the anti-diabetic drug rosiglitazone lead to induction of DC_reg_ (Kalergis et al., 2009). The diureticum triamterene is another compound which might be useful for the therapy of SLE because it is able to induce IDO activity in mDCs and such IDO^+^ DCs have been described to induce fully functional Foxp3 positive CD4^+^CD25^+^ T cells (Ghosh and Branch, 1975; Chung et al., 2009).

### Lack of regulatory DC function is associated with joint inflammation in rheumatoid arthritis and psoriasis

As revealed in murine model systems, DCs play an essential role in keeping immune homeostasis in the joint. Under these conditions, joint derived self-antigens from apoptotic cells can be recognized by DCs via the heterodimeric receptors CR3 (CD11c/CD18) and CR4 (CD11b/CD18) belonging to the β2-integrin family of adhesion molecules (Morelli et al., 2003). Activation of DCs by these receptors induces a regulatory DC phenotype associated with suppressed IL-12 production and elevated secretion of TGFβ. Moreover, it was shown that such DCs are capable of inducing T cell anergy (Morelli et al., 2003).

Rheumatoid arthritis (RA) is a chronic autoimmune disease associated with production of elevated levels of pro-inflammatory cytokines, including IL-12, IL-6, IL-1, IL-23, and TNFα, that are involved in activation of naïve T cells and directing their polarization into T_H_1 and T_H_17 cells. DCs are implicated as major players for the development and persistence of inflammation but also the resulting joint and bone destruction (Table 1). Albeit the number of CD303^+^ pDCs and CD1c^+^ mDCs is significantly reduced (Kavousanaki et al., 2010) in patients with active RA, DCs invading synovial fluids are quickly activated in response to joint-associated factors such as cartilage glycoprotein 39 but also pro-inflammatory stimuli including cytokines and chemokines and other proteins derived from dying cells within synovial fluids (Van Bilsen et al., 2004). DCs in RA patients are also involved in elevated B cell activation followed by proliferation and antibody production. As shown in a murine collagen-induced arthritis model B cell activation seems to be mainly related to the secretion of the cytokine BAFF (B-cell activating factor) which belongs to the TNF family (Zhang et al., 2005). A switch from regulatory to inflammatory functions of DCs together with an overall reduced number of DC_reg_ in RA patients suggests that a reprograming of DCs back to a regulatory phenotype might be an appealing therapeutic strategy for these patients. In fact, higher numbers of pDCs expressing IDO were found in peripheral blood of RA patients following current therapy regimens. These DC_reg_ were reported to convert naïve T cells into IL-10 secreting T_reg_ cells (Kavousanaki et al., 2010). Therefore, a more specific therapeutic attempt in comparison to currently used global immune suppressive drugs, like methotrexate and infliximab, might be to revert back highly activated DCs showing a pro-inflammatory profile into DC_reg_ capable of suppressing autoreactive T cells. Indeed, several early *ex vivo* vaccination trials, like for example the phase I clinical trial Rheumavax at the University of Queensland (Australia) or AutoDECRA at Newcastle University (UK) aim to reestablish T cell tolerance by induction of T_reg_ cells via modification of DC function. Another approach might be to disrupt costimulatory interactions between DCs and T cells. Cytotoxic T lymphocyte antigen-4 (CTLA4) fused to IgG1 interrupts the costimulatory B7/CD28 interaction between T cells and DCs preventing the activation and proliferation of T effector cells (Dall'Era and Davis, 2004). Furthermore, the binding of CTLA4 or its IgG1 fusion molecule was found to induce IDO expression in DC after binding to the B7 surface receptor (Grohmann et al., 2002). Adoptive transfer of CTLA4-Ig treated CD11c^+^ DCs into collagen-induced arthritic mice induced the expansion of CD4^+^CD25^+^Foxp3^+^ T_reg_ cells and prevented the onset of arthritis efficiently (Ko et al., 2010). Another approach to target costimulation was recently achieved by siRNA (short interfering RNAs) technology. DCs loaded with the arthritogenic Ag collagen II and treated with siRNAs targeting the costimulatory molecules CD40, CD80, and CD86 were used in an arthritis mouse model. Treatment with these re-programmed DCs resulted in downregulation of IL-2, IFNγ, TNFα, and IL-17, which in turn resulted in elevated levels of Foxp3^+^ T_reg_ cells (Zheng et al., 2010). Another approach to induce DC_reg_ was recently published demonstrating that dual lentiviral transfer of a constitutive MEK-1 mutant with a fusion protein containing invariant chain of MHC and ovalbumin (IiOVA) leads to activation of ERK signaling thereby inducing TGFβ production by DCs (Arce et al., 2011). As a consequence ERK-activated DCs induced the expansion of Foxp3^+^ T_reg_ cells which prevented joint destruction.

Another example for the lack of DC regulatory function in autoimmunity is psoriasis, one of the most common inflammatory diseases, which is characterized by dermatosis with hyperproliferation of keratinocytes. In this autoimmune disease autoreactive, constitutively activated T cells secrete cytokines which support the proliferation of dermal cells. Genetic, environmental and immunological factors define the severity of psoriasis, but the cause of the disease is still unknown. Psoriasis is also characterized by a reduced number of circulating pDCs while at the same time increased numbers of pDCs are recruited to psoriatic lesions and healthy skin (Skrzeczynska-Moncznik et al., 2009). IFNα itself, but also other soluble mediators like chemerin have been suggested to be responsible for this relocation of pDCs in psoriasis (Skrzeczynska-Moncznik et al., 2009). In the early phase of psoriasis pDCs themselves secrete IFNα subsequently inducing the activation and expansion of T cells (Nestle et al., 2005). Elevated expression of IFNα by DCs in psoriasis might be related to the identification of high level expression of the endogenous anti-microbial peptide LL37 in psoriatic lesions. LL37 can bind to self-DNA resulting in complex structures that activate pDCs via the pattern recognition receptor TLR9 thereby inducing IFNα secretion (Lande et al., 2007). If IFNα is essential for the pathogenesis of psoriasis, its blockade should reduce psoriatic symptoms. Indeed, using blocking anti-IFNα antibodies prevented the development of psoriasis in a xenograft model in mice (Nestle et al., 2005). In addition to IFNα blockade other attempts to reestablish DC_reg_ in psoriasis have been suggested. An interesting strategy to induce DC_reg_ was recently reported by stimulating DCs with the neuropeptide α-melanocyte-stimulating hormone (α-MSH). α-MSH stimulated DCs downregulated the costimulatory molecules CD80 and CD86, upregulated the two coinhibitory molecules PD-L1 and PD-L2, as well as CD205 and secreted high amounts of IL-10. These DCreg subsequently induced CD4^+^CD25^+^Foxp3^+^ T_reg_. Mutation experiments of the MC-1R gene showed that the induction of DC_reg_ was mediated by the binding of α-MSH to the MC-1R and that this effect could ameliorate psoriasis *in vitro* as well as *in vivo* (Auriemma et al., 2012). Another approach to induce DC_reg_ was based on vitamin D. An activated form of vitamin D, namely 1,25-dihydroxyvitamin D3 has been described to inhibit the differentiation and maturation of DCs by its influence on RNA Polymerase II-mediated transcription of response genes (Penna and Adorini, 2000). Downregulation of activation-associated molecules such as CD1a was associated with diminished IL-12 secretion, and upregulation of IL-10 reprogramed DCs toward regulatory functions thereby gaining the capacity to induce T_reg_ cells (Piemonti et al., 2000).

### Myeloid cells are involved in chronic inflammation in obesity

The observation that increased adipose tissue in obesity is associated with the influx of immune cells, mainly myeloid cells has been made less than 10 years ago (Wellen and Hotamisligil, 2003; Chawla et al., 2011). Obesity is associated with Type 2 Diabetes (T2D), insulin resistance, arteriosclerosis, other cardiovascular diseases and even increased cancer risk (Lumeng and Saltiel, 2011). Elevated levels of free fatty acids (FFAs) and pro-inflammatory cytokines are hallmarks of the systemic low-grade inflammation persisting in obese individuals (Roytblat et al., 2000; Hansen et al., 2010). In mouse models massive infiltration of immune cells into adipose tissue has been described suggesting to promote high-fat died induced adipose tissue inflammation (Duffaut et al., 2009; Strissel et al., 2010). While macrophages clearly play a major role during this chronic inflammation the role of DCs in the inflammatory cross-talk of adipocytes and immune cells is not yet clear (Table 1). The impact of dietary fatty acids on the presence of DCs in adipose tissue adjacent to and remote from lymph nodes has been investigated under conditions of chronic mild inflammation induced by low doses of LPS in rats (Mattacks et al., 2004). Chow containing fish oil which is rich in unsaturated fatty acids (USFAs) is considered to reduce inflammatory effects while diets enriched for oils containing saturated fatty acid (SFAs) were associated with an increase of DCs in perinodal adipose tissue (Mattacks et al., 2004). So far, little is known about the role of elevated levels of FFAs on DC function. However, one could speculate that—similar to macrophages—FFAs will shape the functional program of DCs. Using fluorescently labeled palmitic acid, it was recently demonstrated that DCs can uptake fatty acids (Herber et al., 2010). Interestingly, little is known about the accumulation and storage of FFAs in DCs and what role lipid droplets might play in DCs. Increased levels of fatty acids have an impact on antigen processing and presentation. Splenic DCs derived from mice with non-alcoholic fat liver disease (NAFLD), an obesity associated disease, were found to show an impaired processing and presentation of HBsAg in the presence of the saturated fatty acid palmitic acid, but not the unsaturated fatty acid oleic acid (Miyake et al., 2010). Similarly, human blood-derived DCs challenged with antigen demonstrated reduced antigen presentation *in vitro*, lower induction of T cell proliferation and also an induction of the two pro-inflammatory cytokines IL-1β and TNFα in the presence of palmitic acid but not oleic acid (Miyake et al., 2010). Due to the complexity of FFAs concerning saturation, length and isomer structure, it is very likely that mixture of FFAs results in similarly complex immune responses due to differential signal integration. For most FFAs the impact on DC function is not yet understood. One possible receptor for fatty acids is the scavenger receptor CD36 on DCs and various other cells (Silverstein and Febbraio, 2009). But also PRR are involved in the detection of some FFAs. For example, the satured fatty acid lauric acid is recognized via TLR4, one of the major receptors for PPRs on DCs. Recognition of lauric acid via TLR4 was found to upregulate costimulatory molecules CD40, CD80, CD86, MHCII, and to enhance secretion of IL-12 subsequently triggering T cell activation (Weatherill et al., 2005). The opposite effect was shown for DCs stimulated with the omega-3 unsaturated fatty acid docosahexaenoic acid. This fatty acid inhibited LPS-induced upregulation of CD40, CD80, and CD86 as well as MHC class II molecules and prevented T cell activation. Due to these immunoinhibitory effects of unsaturated fatty acids, they might be useful as therapeutics in chronic inflammatory diseases, including obesity, cardiovascular disease, Bowel disease, Crohn's disease, or chronic obstructive pulmonary disease (Blok et al., 1996; Caughey et al., 1996; Calder, 1997; De Batlle et al., 2011; Bassaganya-Riera et al., 2012; Huang et al., 2012). A cis-9, trans-11 isomer of conjugated linoleic acid is a poly-unsaturated fatty acid naturally occurring in meat, milk, and other nutrients. It has been shown to induce the expression of the anti-inflammatory cytokine IL-10 in murine bone-marrow derived DCs in an ERK dependent fashion (Loscher et al., 2005). As negative feedback loop IL-10 inhibited NFκB leading to suppression of its downstream target IL-12 which might contribute to its immunosuppressive properties on T_H_1 cell activation (Loscher et al., 2005). Besides TLR4 also peroxisome proliferation-activator receptor-gamma (PPARγ) can affect the maturation status of DCs (Chinetti et al., 2000; Nencioni et al., 2002). Upregulation of PPARγ was observed in docosahexaenoic acid or eicosapentaenoic acid stimulated bone marrow derived DCs. These cells showed reduced IL-12 secretion, reduced expression of costimulatory molecules, and reduced NFκB activity, but enhanced expression of IL-10R and its ligand IL-10 (Draper et al., 2011). Despite an increased physical interaction and cellular colocalization of NFκB and PPARγ the same study showed that the anti-inflammatory effect of the two tested omega-3 USFAs was not abolished by the inhibition of PPARγ with a chemical inhibitor compound. These results lead to the conclusion, that PPARγ might be an intracellular receptor for unsaturated fatty acids but cannot be singularly made responsible for the inflammatory effects of polyunsaturated fatty acids. Finally, it should be mentioned that resolvins and lipoxins, metabolites of docosahexaenoic acid or eicosapentaenoic acid, have anti-inflammatory functions and are involved in downregulation of IL-12 secretion in DCs via downregulation of the chemokine receptor 5 (CCR5) (Aliberti et al., 2002).

Beneficial usage of omega-3 unsaturated fatty acids has been shown in clinical trials to improve plasma triglyceride levels and insulin activity in T2D patients (Hendrich, 2010). T2D is often associated with obesity and characterized by elevated plasma concentrations of the pro-inflammatory cytokines TNFα and granulocyte-macrophage colony-stimulating factor driving chronic low grade inflammation systemically (Surendar et al., 2012). These two pro-inflammatory mediators contribute to the activation of circulating CD85^+^ CD123^+^ pDCs and CD85^+^ CD33^+^ CD123^dim^ mDCs yet the overall number of DCs is reduced in T2D making diabetic patients prone to infections (Seifarth et al., 2008; Blank et al., 2010). Interestingly, DCs seem to be reprogrammed once concomitant health problems such as arteriosclerosis are acquired. In T2D patients with atherosclerotic complications circulating monocytes and DCs show reduced production of pro-inflammatory mediators like TNFα which is associated with impaired T cell activation (Corrales et al., 2007). Taken together, DCs like macrophages are involved in chronic low-grade inflammation observed in obesity. Elevated levels of free fatty acids, a hallmark of obesity, have substantial influences on DCs with saturated fatty acids inducing pro-inflammatory immune mediators. On the other hand, unsaturated fatty acids seem to suppress inflammation and are currently investigated as an attractive therapeutic option in obesity. Therefore, unsaturated fatty acids constitute yet another mechanism capable of inducing regulatory programs in DCs.

## Regulatory DCs are induced during chronic infections

In general, the immune system is equipped to resolve most acute infections by completely removing invading pathogens. However, some pathogens have evolved strategies to escape a resolving immune response and as a consequence induce chronification of the infection. Chronic infections are also characterized by a deviated immune response often resulting in a prevalence of immunoregulatory mechanisms including regulatory cells. In fact, increasing evidence exists suggesting that DC_reg_ also play a role in chronic infections. In some situations such as the development of chronic granulomatous infections, the development of regulatory mechanisms actually might be the last resort of the host to keep the invading pathogens in check. Using a few examples, we illustrate the evidence for DC_reg_ in chronic infections.

### Deviation of DC function during viral infections

Under normal conditions, RNA and DNA viruses activate DCs e.g., via the PPRs TLR7 or TLR9 resulting in enhancement of IFNα production by matured DCs. In some viral infections lack of viral clearance is—at least in part—due to lack of an effective DC activation and maturation (Table 1). For example although hepatitis C virus (HCV) is recognized by DCs via binding of the HCV envelope 2 protein to surface receptors such as SRBI, DC-SIGN, CD81 or TLR receptors other viral proteins inhibit important steps of DC maturation, prevent IL-12 induction and subsequently reduce their capacity to induce T cell activation (Sarobe et al., 2003). During monocyte differentiation HCV inhibits the expression of CD1a, CD1b, and DC-SIGN, but induces these DCs to secret IL-10 preventing T cell expansion (Tu et al., 2012). Attempts to decipher the molecular mechanism of the immune suppressive pathways activated by HCV revealed that after exposure of pDCs to HCV-infected hepatoma cells they activated IRF7 but not the central pro-inflammatory transcription factor NFκB. These finding might explain, why pDCs in HCV infection secrete IFNα but are not able to produce IL-6 or TNFα (Dental et al., 2012). In mice, the function of DCs was recovered when DCs were pulsed *ex vivo* with the viral protein NS3 and matured in the presence of CpG (Yu et al., 2006). Unlike HCV infection, the capability to secrete IL-12 is not impaired in herpes simplex virus type 1 (HSV1) infected DCs. In an initial report, DCs infected with HSV1 were reported to show many phenotypical features of matured DCs except for a loss of CD83 and reduced T cell stimulating activity (Kruse et al., 2000). A more recent study however demonstrated in a murine model that the HSV1 latency-associated transcript (LAT) is involved in the downregulation of costimulatory molecules and pro-inflammatory cytokines like IL-6, IL-12, and TNFα (Chentoufi et al., 2012). Impaired DC function was also suggested to contribute to the reduced function of HSV-specific CD8^+^ T cells (Chentoufi et al., 2012). Moreover, LAT can function like endogenous cFLIP inhibiting caspase-8 mediated apoptosis in HSV-infected DCs thereby ensuring spreading and replication of the virus (Kather et al., 2010). Yet other mechanisms of immune deviation operative in DCs have been suggested for human immunodeficiency virus (HIV) infection. In the early phase of primary HIV infection, numbers of CD11c^+^ mDCs as well as CD123^+^ pDC are decreased resulting in a lack of anti-viral IFNα expression (Pacanowski et al., 2001). Infected DCs display a rather immature phenotype because they do not really upregulate typical activation markers like MHC class II, CD83, CD80, CD86, or CCR7 (Smed-Sorensen et al., 2004; Martinson et al., 2007). Their functional potential is controversially discussed. Although the number of circulating DCs is declinedin HIV patients, the remaining DCs seem to keep a certain immune activating potential as exemplified by CD86 upregulation after stimulation with CD40L. Also these DC secrete TNFα, IL-1β, and IL-10 but do not secrete IL-12 p70 or IFNα (Smed-Sorensen et al., 2004; Martinson et al., 2007). The induction of IFN regulatory factor 1 (IRF-1) by HIV infection of DCs was shown to induce IFN-stimulated genes without the expression of type I and II interferons thereby ensuring the replication of the HIV in mDCs (Harman et al., 2011). In fact, the loss of IFNα production of HIV infected pDCs was postulated to contribute to progression of the disease (Tilton et al., 2008). Furthermore, HIV infected DCs are resistant to NK cell mediated cell death. Melki and coworkers showed an upregulation of the anti-apoptotic molecules c-FLIP and c-IAP2 in these DCs rendering them resistant to TRAIL-induced cell death via interaction between TRAIL on the cell surface of NK cells and its receptor DR4 on DCs (Melki et al., 2010).

These are only few examples, demonstrating that the induction of DCs with regulatory rather than stimulatory functions is a major theme of many viruses capable of escaping an efficient immune response thereby inducing viral persistence.

### DC_reg_ are essential players in chronic granulomatous listeriosis

Myeloid cells with regulatory function have also been implicated in the formation and maintenance of granulomatous structures that are supposed to enclose pathogens thereby preventing their dissemination (Table 1). While macrophages play the major role in most granulomatous diseases chronic granulomatous listeriosis is characterized by large quantities of DCs within the cellular ring wall of the granuloma (Popov et al., 2006). Listeriosis is caused by the gram positive bacteria *Listeria monocytogenes (L.m.)*. In the immunocompetent host, clinical manifestation is characterized by a self-limiting gastroenteritis. However, in immunocompromised individuals, but also elderly people and newborns *L.m.* can cause chronical meningoencephalitis and septicemia (Swaminathan and Gerner-Smidt, 2007; Allerberger and Wagner, 2010; Mook et al., 2011). Moreover, advanced stages of chronic disease are characterized by granulomas in lymph node tissue (Gray and Killinger, 1966). Granulomas are organized immune cell aggregates that form in response to persistent stimuli of infectious or non-infectious nature (Ramakrishnan, 2012). Recently, our group discovered that CD11c^+^ S100^+^ IDO^+^ mDCs are predominant constituent elements in the outer wall of listeria granuloma (Popov et al., 2006, 2008; Von Bergwelt-Baildon et al., 2006). Transcriptome profiling of *L.m.* infected mDCs revealed that infection of mDCs leads to the upregulation of numerous effector molecules including TNFα, IFNγ, IL-10, COX-2, IDO, and CD25 that act together in a regulatory fashion (Popov et al., 2008). Supernatants derived from mDCs infected by *L.m.* suppressed T cell proliferation via a mechanism that involves IDO activity, (Popov et al., 2008 and Schultze, unpublished data). Furthermore, IDO^+^ mDCs were also capable to keep *L.m.* in check, suggesting that IDO-competent mDC possess microbicidal activity (Popov et al., 2006, 2008; Von Bergwelt-Baildon et al., 2006) and Nino-Castro, unpublished results). These regulatory DC seem to have at least three critical roles during chronic listeriosis. First, they are involved in the formation and maintenance of the granulomatous structure, second, they keep the pathogen in check, and third, they suppress T cell effector function that could destroy the granulomatous structure. Therefore, these DC_reg_ are essential for the formation and maintenance of this immune priviledged site (Mellor and Munn, 2004) at the same time acting as a barrier that controls bacterial growth and dissemination via an IDO mediated mechanism.

### DC_reg_ are important cells in numerous parasitical infections

Despite their phylogenetic diversity, parasitic protozoans and helminthes have the ability in common to produce long lasting chronic infections (Peters and Sacks, 2006). Understanding how parasitical infections can progress toward chronicification poses a challenge due to the complex host pathogen interactions that depend on the immunological status of the host, but also on the genetic background of both pathogen and host. Some evidence suggests a role for DCs with regulatory function during infection with parasites.

Species of the genus *Leishmania* are the causing agents of leishmaniases, a group of illnesses of the oral and respiratory mucosae, the skin, and the reticuloendothelium (Reithinger et al., 2007). The cutaneous form is the most common one and is also known to evolve into a chronic disease. Recently, it has been reported in a murine model, that a subset of Langerin^+^ Langerhans cells might favor the recruitment of T_reg_ cells to the site of infection resulting in aggravated disease. This DC subset was able to promote T_reg_ expansion *in vitro* (Kautz-Neu et al., 2011). Moreover, it has been reported that mice infected with *Leishmania major* upregulate IDO expression in lymph node pDCs. IDO^+^ pDCs suppressed T cell activation and proliferation *in vitro*. Furthermore, the inhibition of the IDO enzymatic activity resulted in an improvement of the immune response toward *L. major* manifested by a reduction in lesion size and parasitical burden (Makala et al., 2011). Although IDO has a well-known microbicidal role by limiting pathogen spread via Trp depletion, the evidence in *Leishmania* infection suggest that in this context IDO^+^ pDCs are deleterious to the host, due to their capacity to attenuate adaptive immune responses. Finally, Nguyen and coworkers reported that infection of murine bone marrow stromal cells by *Leishmania donovani* enhanced their capacity to attract hematopoietic progenitor cells. *L. donovani* infected stromal cells were able to support the development DCs from hematopoietic progenitor cells. Moreover, this DC subset acquired regulatory properties being able to suppress CD4^+^ T cell proliferation *in vitro* (Nguyen Hoang et al., 2010). In summary, there is sufficient evidence that different DC populations can acquire regulatory functions in response to *Leishmania* infection, however their exact role during chronification of the disease is not yet fully understood.

Another example for the involvement of DC_reg_ in parasite infections is Chagas disease caused by the parasite *Trypanosoma cruzi.* In most patients with Chagas disease an immune response develops, the parasitemia wanes, and signs and symptoms resolve completely within a few months. However, around 30% of the patients will progress toward a chronic disease (Hemmige et al., 2012). The mechanisms that control the progression of the disease into a chronic phase are widely unknown (Sathler-Avelar et al., 2009). Recently, it has been reported that coculture of DCs with *T. cruzi trypommastigotes in vitro* leads to production of IL-10 and TGFβ by DCs. Furthermore, these cells suppressed T cell proliferation *in vitro* (Poncini et al., 2008), which indicates that *T. cruzi* might induce DCs with regulatory functions to evade the host immune system. However, to our knowledge there is no evidence yet supporting the role of DC_reg_ as promoters of tolerance toward *T. cruzi* in infected hosts *in vivo*. Therefore, the potential role of DC_reg_ in the overall immune response against this pathogen remains unclear. Nematode infections are known to promote local immunosuppression in the host, allowing the parasite to achieve a long term survival, which is usually associated with a local chronic infection (Taylor et al., 2005). Recently, Li and coworkers described a naturally occurring DC subset with regulatory activity in the murine infection model of *Heligmosoides poligyrus.* These CD11c^low^ CD45^mid^ DC_reg_ expanded rapidly after *H. poligyrus* infection and promoted T_reg_ differentiation *in vitro* (Li et al., 2011).

Taken together, chronification of infectious diseases is very often accompanied by the induction of DCs with regulatory functions. Reprogramming these APC away from immunostimulatory functions seems to be a central theme during the chronification process. However, more work is required to really understand the mechanisms that lead to the observed reprogramming of DCs.

## Summary and conclusions

During the last two decades immunostimulatory functions of DCs were the major focus of DC research (Steinman, 2007) consolidating that these extraordinary cells are the main contributors for immune activation. However, during the last years accumulating evidence strongly suggests that DCs are much more versatile in their functions allowing them to regulate (DC_reg_) or even halt immune responses e.g., by inducing tolerance (tolerogenic DC). DC_reg_ are implicated in terminating inflammatory responses under physiological conditions yet regulatory DC functions also seem to be hijacked by pathologic conditions as different as cancer or chronic infection. While several of the molecules associated with regulatory DC function (IL-10, TGFβ, IDO, PGE_2_, and CD25) have been elucidated and observed in numerous cancer entities and chronic infections, the molecular mechanisms reprogramming DC to become DC_reg_ are still poorly understood. Many previous studies are rather descriptive and many findings in murine and rodent models often could not be translated to the human, leaving researchers confronted with the problem to investigate molecular mechanisms of DC_reg_ involvement in human specimens. However, the availability of sequencing-based genomic technologies assessing networks of epigenetic, transcriptional, and translational regulation on a genome-wide scale and in an unprecedented resolution makes research into human DC_reg_ biology a very promising endeavor. We envision that the integration of such data will allow us in the future to better understand how exogenous signals are integrated in DCs to foster a regulatory rather than an immunostimulatory program. Moreover, signal integration is not only restricted to classical pro- and anti-inflammatory mediators but is further altered by a myriad of soluble and cell-contact mediated signals as exemplified here for metabolites like kynurenines or unsaturated fatty acids. Moreover, understanding DC_reg_ biology will also guide the development of better DC-based vaccines, e.g., as cancer immunotherapy. Unraveling differential and context-dependent signal integration by DCs during activation will also lead to the discovery of genes that could be targeted for immunomodulation.

### Conflict of interest statement

The authors declare that the research was conducted in the absence of any commercial or financial relationships that could be construed as a potential conflict of interest.
